# A Laminar Organization for Selective Cortico-Cortical Communication

**DOI:** 10.3389/fnana.2017.00071

**Published:** 2017-08-22

**Authors:** Rinaldo D. D’Souza, Andreas Burkhalter

**Affiliations:** Department of Neuroscience, Washington University School of Medicine St. Louis, MO, United States

**Keywords:** cortical hierarchy, mouse visual cortex, interareal communication, layer 1, cortical inhibition

## Abstract

The neocortex is central to mammalian cognitive ability, playing critical roles in sensory perception, motor skills and executive function. This thin, layered structure comprises distinct, functionally specialized areas that communicate with each other through the axons of pyramidal neurons. For the hundreds of such cortico-cortical pathways to underlie diverse functions, their cellular and synaptic architectures must differ so that they result in distinct computations at the target projection neurons. In what ways do these pathways differ? By originating and terminating in different laminae, and by selectively targeting specific populations of excitatory and inhibitory neurons, these “interareal” pathways can differentially control the timing and strength of synaptic inputs onto individual neurons, resulting in layer-specific computations. Due to the rapid development in transgenic techniques, the mouse has emerged as a powerful mammalian model for understanding the rules by which cortical circuits organize and function. Here we review our understanding of how cortical lamination constrains long-range communication in the mammalian brain, with an emphasis on the mouse visual cortical network. We discuss the laminar architecture underlying interareal communication, the role of neocortical layers in organizing the balance of excitatory and inhibitory actions, and highlight the structure and function of layer 1 in mouse visual cortex.

## Introduction

The neocortex, arguably the pinnacle of mammalian evolution, is a layered sheet that blankets the forebrain. It is critically involved in sensory perception, guiding actions, paying attention and interpreting the world around us (Cauller, 1995; Treisman, 1996; Alfano and Studer, 2013). To perform these functions, neocortical circuits must selectively extract and amplify neuronal signals that encode various features of sensory stimuli, compare incoming signals with stored information, and route them to specialized circuits both within and outside the cortex (Douglas and Martin, 2007; Shipp, 2007; Harris and Mrsic-Flogel, 2013; Harris and Shepherd, 2015). Excitatory projection neurons and diverse local inhibitory interneurons in the neocortex form an intricate network in which synaptic connections between the neurons reveal a high level of specificity (Binzegger et al., 2004; Jiang et al., 2015). This specificity includes the genetic identity of the source and target neurons, the cortical areas the neurons reside in, and the precise locations of inputs on a neuron’s dendrites (Groh et al., 2010; Sorensen et al., 2015; Zeisel et al., 2015; Tasic et al., 2016; Feldmeyer et al., 2017). The network includes local circuits composed of neurons within tens of microns of each other, as well as long-range pathways that interconnect areas that are millimeters or centimeters apart. Each cortical projection neuron consequently receives inputs from thousands of other neurons (Elston et al., 2009); the timing, strength and polarity (i.e., whether inhibitory or excitatory) of these inputs together with the intrinsic membrane properties of the postsynaptic cell (reviewed in Whitmire and Stanley, 2016) determine the projection neuron’s spike output. The high specificity of connections results in a variety of functional motifs including recurrent excitation (Douglas et al., 1995; Douglas and Martin, 2007), feedforward inhibition (see Box 1; Pouille et al., 2009; Isaacson and Scanziani, 2011), and divisive and subtractive normalization caused by counterbalanced inhibition (Carandini and Heeger, 2011; Wilson et al., 2012), each of which plays important, specific roles in signal amplification and gain control.

Box 1 Note that each of the words *feedforward* and *feedback* has two distinct meanings in this manuscript. When classifying pathways or axonal projections, the words describe the direction of signal flow within a hierarchy. On the other hand, feedforward and feedback *inhibition*, is a circuit motif whose definition is independent of pathway or hierarchy. For example, feedforward inhibition can be generated within both feedforward and feedback pathways.

Further constraining the diversity of synaptic inputs that each neuron receives is the cortex’s layered architecture, commonly identified by the size and density of neurons and the arrangement of afferent inputs. As a result, a major determinant of the output of a neuron is its laminar location as well as the shape and extent of its dendritic tree (DeFelipe and Fariñas, 1992; Major et al., 2013). For instance, synaptic inputs to distal regions of a pyramidal cell’s apical dendrite would be substantially more attenuated at the cell body than inputs to more proximal sites (Stuart and Spruston, 1998; Williams and Stuart, 2002). As a result, projection neurons must integrate a temporal pattern of postsynaptic currents of varying amplitudes, leading to a spike readout that is a result of nonlinear summations of synaptic inputs from different layers (Spruston, 2008). The laminar organization of neurons and their afferents, both long-range and local, is therefore central to neocortical function (Douglas and Martin, 2004). In this manuscript, we review studies that have provided important insights into the laminar structure of hierarchically organized cortico-cortical networks and discuss how the interplay between excitation and inhibition within the different laminae may differentially regulate signal transmission through intracortical and cortico-thalamo-cortical pathways.

## Anatomy of Cortical Hierarchy

The task of processing the diverse features of a sensory stimulus within the neocortex is distributed across a mosaic of many distinct, interconnected *areas* that are characterized by distinct connectivity profiles, cytoarchitecture, functions and developmental specification (Felleman and Van Essen, 1991; Andermann et al., 2011; Marshel et al., 2011; Alfano and Studer, 2013; Glasser et al., 2016). In non-human primates the areas involved in vision and visually guided actions can be described formally as being in a distributed hierarchical network with areas higher up the hierarchy underlying the representation of increasingly complex features of visual stimuli (Maunsell and van Essen, 1983; Felleman and Van Essen, 1991; Markov and Kennedy, 2013; Laramée and Boire, 2014). Visual signals are transmitted from lower to higher areas through so-called *feedforward* pathways (Box 1) that typically project in the rostral direction initiating from the posterior-most primary visual cortex (V1; Bastos et al., 2012; Markov and Kennedy, 2013). Concurrently, caudally-projecting sensory and motor *feedback* pathways are thought to be involved in contour integration of local stimulus features, making predictions of sensory stimuli, resulting in the context-dependent selection and modulation of relevant feedforward inputs (Bastos et al., 2012; Larkum, M. 2013; Saleem et al., 2013; Vaiceliunaite et al., 2013; Chen et al., 2014; Pafundo et al., 2016; Pakan et al., 2016; Attinger et al., 2017; Kuchibhotla et al., 2017; Nandy et al., 2017). This has led to the suggestion that ascending signals encode errors between the expected (predicted) and the actual response to sensory input, a mechanism referred to as predictive coding (Rao and Ballard, 1999; Bastos et al., 2012; Shipp, 2016).

Because of their divergent functions in bottom-up and top-down processing, it is perhaps not surprising that feedforward and feedback pathways exhibit anatomical differences across species. Felleman and Van Essen (1991) famously constructed a hierarchy of the macaque monkey visual cortex by examining termination patterns of cortico-cortical axonal projections from hundreds of prior studies and by classifying these pathways as being feedforward, feedback, or lateral (i.e., connecting areas at the same level of a hierarchy). In this classification, projections that were densest in layer 4, but which often included other layers as well, were considered feedforward; pathways preferentially terminating in superficial and deep layers were classified as being feedback; and pathways that terminated more uniformly in all layers were described as being lateral (Rockland and Pandya, 1979; Maunsell and van Essen, 1983; Felleman and Van Essen, 1991). While this meta-analysis has been extremely influential in our understanding of coding mechanisms within hierarchical networks, studies in non-primate animal models have shown that the exact laminar patterns formed by ascending and descending interareal projections differ between species. In the adult cat, for example, projections from V1 to higher cortical areas 18 and 19 terminated strongest in layers 2/3, with substantially weaker inputs to layer 4, although V1 projections to the medial bank of the suprasylvian sulcus had a more primate-like feedforward appearance with strongest terminations in layer 4 (Price and Zumbroich, 1989). This is noteworthy because Felleman and Van Essen (1991) regarded projections in the macaque cortex that were densest outside of layer 4 to be descending. Similarly in rat, axons from V1 to higher visual areas showed a multilaminar organization with roughly equally dense terminations in layers 2–5 (Coogan and Burkhalter, 1990, 1993), reminiscent of the description of lateral connections in macaque (Felleman and Van Essen, 1991). These feedforward laminar termination patterns were distinct from feedback terminations, which were densest in layers 1 and 6 (Coogan and Burkhalter, 1990, 1993).

The differences in lamination patterns of interareal connections may be expected based on the diversity of laminar architectures and mRNA expression profiles across species; for example, V1 in primates can be divided into twelve rather than six cortical layers commonly annotated in rodents (Belgard et al., 2011; Bernard et al., 2012; Balaram and Kaas, 2014). An important contributing factor for this diversity in cortical lamination is the difference in proliferative cell cycles during corticogenesis in different species, particularly the role of the outer subventricular zone in the expansion of the superficial layers in primate cortex (Lui et al., 2011; Dehay et al., 2015). Thus the species-specificity of laminar patterns may reflect the disparate organizations of circuits required for network processing adapted to species-variant properties of cortices such as brain size, number of areas, network density and the ecological niche within which the animals evolved to survive and thrive (Kaas, 2013; Laramée and Boire, 2014). A preserved feature across mammals, however, is that feedforward connections terminate most densely in layers 3 and 4. In contrast, feedback projections are densest in layer 1, which is less strongly innervated by local, lateral and feedforward connections (Thomson and Bannister, 2003; Binzegger et al., 2004; Shipp, 2007).

With the development of powerful tools for identifying, recording and manipulating neuronal circuits with unprecedented resolution and accuracy, the mouse has emerged as an extremely useful model to examine the organization, function and synaptic architecture of the mammalian visual system (Havekes and Abel, 2009; Huberman and Niell, 2011; Katzner and Weigelt, 2013). Constructing the mouse visual cortical hierarchy is therefore an important step in the study of visual function. Based on the laminar termination patterns of interareal axonal afferents within the mouse cortical network, the density of interareal projections in layers 2–4 relative to that in layer 1 was analyzed to show a clear hierarchy between three areas, V1, LM (the lateromedial area), and PM (the posteromedial area; D’Souza et al., 2016). The relative hierarchical positions of the three areas were consistent with the increase in their respective receptive field sizes (Wang and Burkhalter, 2007). The axonal termination patterns in the higher areas suggest that layers 2–4 in mouse neocortex plays the role of the primate middle layers as the primary target of feedforward afferent connections. Supporting this idea is the observation that geniculocortical afferents to V1, while densest in layer 4, also terminate in layers 1–3 (Antonini et al., 1999; Cruz-Martín et al., 2014). The interareal connection from LM to PM also indicates that layer 1 may be an important target of feedforward projections originating in higher areas (D’Souza et al., 2016). The complete hierarchy of the approximately ten to sixteen areas that make up the mouse visual cortical network (Wang and Burkhalter, 2007; Andermann et al., 2011; Marshel et al., 2011; Garrett et al., 2014; Zhuang et al., 2017) is yet to be determined.

The anatomical hierarchy of visual cortex is observed not only in the organization of interareal axonal terminations, but also in the laminar locations of the cell bodies from which they originate (Maunsell and van Essen, 1987; Markov and Kennedy, 2013). In order to obtain a quantitative measure for hierarchical levels, the primate cortical hierarchy was constructed by measuring the proportion of neurons in layers 2 and 3 that project to a target area, to the total number of projecting neurons (Barone et al., 2000; Markov et al., 2014). The analyses were based on the observation that in primates, the fraction of supragranular neurons that project to a target area depends not only on whether the projections were feedforward or feedback, but also on the hierarchical distance between the two areas (Barone et al., 2000).

Somewhat surprisingly, given the striking organization in the primate brain, no such laminar segregation of source neurons projecting through feedforward and feedback pathways was observed in the mouse visual cortex (Berezovskii et al., 2011). By injecting retrograde tracers into V1 and the anterolateral area AL of adult mice, the authors of this study showed that LM neurons that projected to a lower area (V1) and those that projected to a higher area (AL) were both found intermingled predominantly in layers 2–4, with no obvious laminar separation. Despite the lack of laminar separation of feedforward and feedback source neurons, only a very small proportion of individual neurons in mouse V1 projected in both feedforward and feedback directions, with the vast majority projecting either only to V1 or to AL (Berezovskii et al., 2011), indicating a segregation of neurons depending on their target areas, similar to what has been observed in the macaque cortex (Sincich and Horton, 2003; Markov et al., 2014). This implies that, except for a tiny minority, individual pyramidal neurons that project to another area (these do not include the corticothalamic pyramidal cells of layer 6; Harris and Shepherd, 2015) can broadly be classified as being either feedforward- or feedback-projecting. These two putative populations of pyramidal neurons may differ in their dendritic morphologies with apical tufts in layer 1 more common in feedforward-projecting neurons (Markov et al., 2014), suggesting pathway-differences in the integration of synaptic inputs to layer 1.

## The Cortico-Thalamic-Cortical Pathway

In parallel with the cortical hierarchy within which areas communicate directly with each other, an additional, commonly observed mode of cortico-cortical communication is via a transthalamic route in which a higher-order thalamic nucleus relays information from one cortical area to another (reviewed in Sherman, 2017). In such a cortico-thalamic-cortical pathway, cortical layer 5 pyramidal cells from one area project their axons to the thalamus where they provide “driver” inputs (strong inputs that activate ionotropic glutamate receptors on proximal dendrites; Sherman and Guillery, 1998) to thalamic relay cells, which themselves project to another cortical area. These driver inputs are in contrast to “modulator” glutamatergic inputs, which have distinct synaptic properties and are thought to modulate the responses to driver glutamatergic inputs, much like the actions of “classic” neuromodulators such as acetylcholine and serotonin (Sherman and Guillery, 1996, 1998, 2011). In the visual system, the pulvinar is a higher-order thalamic nuclei that receives inputs from, and sends afferents to, a number of visual cortical areas, and is therefore a key hub for visual cortico-cortical communication (Sherman and Guillery, 1996; Grieve et al., 2000; Shipp, 2003). In the mouse, the lateral posterior nucleus (LP; the rodent analog of the pulvinar), likely mediates transthalamic cortico-cortical information flow, receiving inputs from layers 5 and 6 of V1 and transmitting signals to (as well as receiving signals from) higher visual areas (Oh et al., 2014; Tohmi et al., 2014; Roth et al., 2016). LP also projects diffusely to layer 1 of V1 providing locomotion-related information (Roth et al., 2016).

Results from a number of studies indicate that the axons of cortical layer 5 neurons, in addition to providing input to the thalamus, branch out to innervate other parts of the brain including midbrain and pontine areas (Deschênes et al., 1994; Bourassa and Deschênes, 1995; Bourassa et al., 1995; Kita and Kita, 2012; Sherman, 2017). This suggests that an identical message, originating in a single axon, is transmitted to a number of different structures that underlie both sensory and motor functions. It has been proposed, therefore, that a crucial function of layer 5 pyramidal neurons that underlie visual cortico-thalamic-cortical communication, but which also branch their axons to other motor structures, is to generate the *efference copy*, a type of neuronal message that helps an animal perceive the environment as being stable even while it moves around in it (Wurtz et al., 2011; Sherman, 2017).

## Distinct Excitation/Inhibition Balance within Laminae

**Figure 1:**
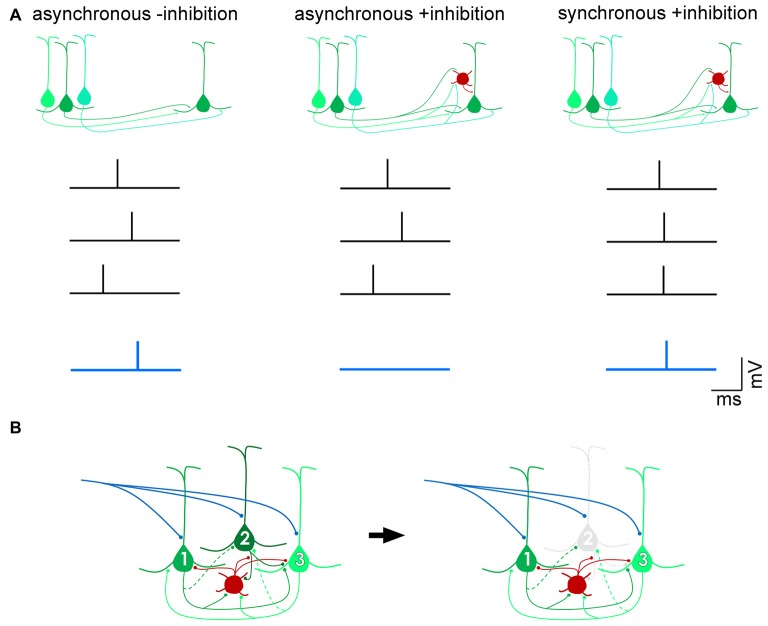
Distinct mechanisms of gain control by feedforward and feedback inhibition. **(A)** Feedforward inhibition. In the absence of inhibition, excitatory postsynaptic potentials (EPSPs) arising from three presynaptic action potentials (black traces) that arrive within a broad time window (“asynchronous -inhibition”) can summate to drive the postsynaptic pyramidal cell past threshold and fire an action potential (blue trace). In the presence of a feedforward inhibitory circuit (red, interneuron), the EPSPs are unable to successfully drive the cell past spike threshold (“asynchronous +inhibition”), unless they arrive within a narrow time window (“synchronous +inhibition”). In this way, a feedforward inhibitory mechanism allows for only coincident inputs to transmit signals, filtering out asynchronous “noise”. **(B)** Feedback inhibition. Pyramidal neurons 1 and 3 are more strongly, reciprocally connected with each other than with pyramidal neuron 2 (dotted axon indicates weak input). The interneuron (red) is reciprocally connected with all three pyramidal neurons. Upon onset of an excitatory input (blue), recurrent excitation between pyramidal neurons 1 and 3 is strong enough to overcome feedback inhibition from the interneuron. Pyramidal neuron 2, however, is inhibited and does not contribute to computations performed by the circuit. Such a motif dynamically alters the components of the circuit depending on permutations of recurrent connections between pyramidal cells and inhibitory interneurons (Douglas and Martin, 2009).

The importance of balanced inhibitory control of excitatory drive within and between cortical areas has been widely reported (Shadlen and Newsome, 1998; Douglas and Martin, 2009; Isaacson and Scanziani, 2011; Whitmire and Stanley, 2016). In a number of cortical areas, inhibition has been shown to scale with excitation (Okun and Lampl, 2008; Xue et al., 2014; Zhou et al., 2014), in order to sharpen receptive fields (Wehr and Zador, 2003), restrain recurrent excitation (Douglas and Martin, 1991; Sanchez-Vives and McCormick, 2000; Pinto et al., 2003), and preserve the temporal fidelity of cortical output (Pouille and Scanziani, 2001; Pouille et al., 2009). By modulating the gain of excitatory projection neurons, inhibitory neurons maintain a wide dynamic range over which brain circuits can effectively respond to sensory stimuli without saturating spike firing (Shadlen and Newsome, 1998; Pouille et al., 2009). Feedforward inhibitory (Box 1) control can occur by inducing pyramidal cells to act as coincidence-detectors so that only excitatory postsynaptic currents (EPSCs) resulting from spikes that arrive within a narrow time window would be permitted to summate and generate spikes in the target neuron and subsequently transmit salient information (Figure 1A). Such a mechanism allows for precise computations of input signals within noisy regimes wherein cortical neurons are continuously bombarded with hundreds or even thousands of inputs per second (Shadlen and Newsome, 1998; Kremkow et al., 2010; Bruno, 2011). In addition to signal transmission governed by feedforward inhibition, gain control can also be achieved by feedback inhibition (Box 1) within highly recurrent networks (Douglas et al., 1995; Douglas and Martin, 2007). In circuits dominated by strong recurrent, excitatory connections that amplify weak, e.g., thalamocortical, inputs (Douglas et al., 1995; Lien and Scanziani, 2013), the feedback inhibitory motif has been proposed to non-linearly modulate cortical gain by silencing individual pyramidal cells, thus transiently reconfiguring local excitatory circuits by selectively eliminating the excitatory components of a winner-take-all network (Douglas and Martin, 2009; Rutishauser et al., 2015; Figure 1B). Another proposed mechanism of gain control is through the balanced increase in excitatory and inhibitory background activity leading to an increase in the membrane conductance of neurons (Chance et al., 2002). Because spontaneous activity is thought to primarily be dependent on cortico-cortical connections (Sanchez-Vives and McCormick, 2000; Timofeev et al., 2000), which have a pathway-specific laminar profile (Binzegger et al., 2004), the modulation of cortical gain is likely to be layer-specific.

If an important property of cortical lamination is the segregation of functionally diverse pathways specialized for distinct spatiotemporal stimulus features (Nassi and Callaway, 2009), it would be reasonable to predict contrasting relative levels of excitation and inhibition in different layers. *In vivo* recordings from a number of studies suggest this to be true. Neurons in different layers of mouse neocortex have been shown to differentially represent sensory cues, particularly through the “sparseness” of cortical activity, in a number of areas (Barth and Poulet, 2012; Harris and Mrsic-Flogel, 2013; Petersen and Crochet, 2013). For mice performing a whisking task, recordings from barrel cortex suggested an overall sparse representation of stimuli (10% of neurons responsible for approximately 50% of all recorded spikes, and 50% of neurons contributing to less than 3% of spikes), with the largest proportion of silent neurons in layer 2/3 (O’Connor et al., 2010). The median firing rates of neurons recorded in this study were highest in layers 4 and 5, and lowest in layers 2/3 and 6. Extracellular recordings in mouse V1 showed that excitatory neurons in layers 2/3 and 4 exhibit a substantially lower rate of spontaneous spiking activity, and have smaller receptive field sizes, than neurons in layers 5 and 6 (Niell and Stryker, 2008). Similarly in auditory cortex, pyramidal neurons in layers 2/3 showed a much sparser level of activity, both evoked and spontaneous, than the deeper layer 5 cells (Sakata and Harris, 2009). A major contributor to the emergence of sparse coding, i.e., the observation that only a few active neurons underlie the representation of a sensory stimulus, is the strong inhibitory actions of local interneurons (Crochet et al., 2011; Haider et al., 2013; Harris and Mrsic-Flogel, 2013; Petersen and Crochet, 2013). These observations therefore indicate a higher level of inhibitory drive to superficial pyramidal neurons compared to those in the deep layers.

Consistent with the observed laminar differences in neuronal activity, results from synaptic and circuit-level studies further point to layer-specific differences in the relative levels of excitation and inhibition. In the mouse primary auditory cortex, for example, the balance between excitatory and inhibitory inputs showed a layer-dependence such that while the amplitudes of inhibitory postsynaptic currents (IPSCs) scaled with those of EPSCs in response to varying intensities of an auditory tone, the excitation/inhibition balance was scaled down in layer 2/3, but was unchanged in layer 4, during behavior (Zhou et al., 2014). In the hindlimb somatosensory cortex, interhemispheric input could evoke inhibition to the distal dendrites of layer 5 pyramidal neurons, but not to pyramidal neurons residing in layer 2/3, indicating distinct regulation of excitation/inhibition balances in the different layers by callosal projections (Palmer L. M. et al., 2012).

**Figure 2:**
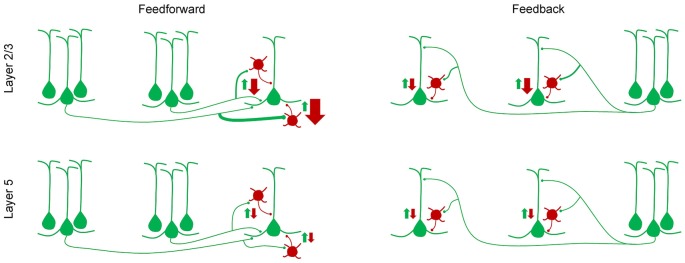
A simplified model of how feedforward inhibition varies with hierarchical distance, pathway and laminar location of target neurons (D’Souza et al., 2016). In layer 2/3, the strength of interareal excitation of parvalbumin (PV) interneurons, relative to that of pyramidal cells, shows a gradual decline from the most feedforward to the most feedback pathway, i.e., from the pathway with the largest hierarchical distance in the feedforward direction, to the pathway with the largest hierarchical distance in the feedback direction. Hierarchical distances were quantified by measuring the ratio of the density of axonal projections in layers 2–4 to that in layer 1. In layer 5, no such gradient in the inhibition/excitation balance is observed and the overall relative excitation of PV interneurons is lower than in layer 2/3.

Similarly, laminar differences in synaptic inputs to excitatory and inhibitory neurons were also observed in the visual cortex. Within the mouse visual cortical network, the primary neuronal targets of feedforward and feedback connections between areas are pyramidal cells and the parvalbumin-expressing (PV+) GABAergic interneurons (Gonchar and Burkhalter, 1999, 2003). The strength of these interareal connections was shown to depend on pathway and on the postsynaptic cell type: interareal excitatory synaptic input to PV+ interneurons was stronger than that to pyramidal neurons in most pathways terminating in layer 2/3 but not in layer 5 (Yang et al., 2013; D’Souza et al., 2016). Further, within layer 2/3, the interareal excitation of PV+ interneurons, relative to that of pyramidal cells, showed a gradual decrease from the most feedforward to the most feedback pathway (Figure 2). Because PV+ interneurons are a major source of inhibition in the neocortex, inhibiting neighboring pyramidal cells with high probability (Yoshimura and Callaway, 2005; Packer and Yuste, 2011; D’Souza et al., 2016), these results suggest that the highest levels of interareal inhibition of pyramidal cells are driven by ascending pathways projecting to higher cortical areas. Notably, the hierarchical dependence of inhibition was not seen in layer 5 neurons where relative targeting of PV+ interneurons was similar across the hierarchy and was generally lower than in the upper layers (Yang et al., 2013; D’Souza et al., 2016).

Layer 2/3 consists of networks characterized by strong recurrent excitatory connections, which have been implicated in selectively amplifying salient inputs within a noisy regime that match information stored in the weights of excitatory synaptic connections (Douglas and Martin, 2007). Therefore, stronger inhibition in the superficial layers suggests that more effective control is required to counterbalance and dynamically regulate excitatory networks within these layers (Douglas and Martin, 2009). Because a canonical function of layer 2/3 pyramidal cells is to convey spike-encoded information to other cortical areas, the stronger targeting of PV+ interneurons may protect against signal corruption across the hierarchical cascade. This is particularly important because pyramidal cells in higher areas show an increasingly higher number and density of dendritic spines (implying a larger number and density of excitatory inputs impinging on them; Elston, 2003; Benavides-Piccione et al., 2006; Elston et al., 2006; Gilman et al., 2017), and integrate inputs over a broader time window (Murray et al., 2014; Chaudhuri et al., 2015).

Lower levels of PV+ interneuron recruitment in layer 5 supports the notion that pyramidal cells within this layer, particularly the subpopulation that projects to subcortical targets, use a “dense coding” strategy to transmit signals (Sakata and Harris, 2009; Harris and Mrsic-Flogel, 2013). These so-called pyramidal tract (PT) neurons are restricted to layer 5, are characterized by thicker apical dendrites and larger cell bodies, and project their axons outside of the telencephalon (neocortex and striatum) to targets that include the brainstem, superior colliculus, spinal cord and higher-order thalamus (Sakata and Harris, 2009; Harris and Mrsic-Flogel, 2013; Shepherd, 2013; Harris and Shepherd, 2015), putatively mediating cortico-thalamic-cortical communication and generating an efference copy (Sherman, 2017) as described in the previous section. It has been proposed that a dense coding strategy in which a relatively large number of neurons respond to a sensory stimulus, and with relatively high firing rates, allows for efficient transmission of signals to distant targets while minimizing the physical volume of neurons and their fibers (Harris and Mrsic-Flogel, 2013). This is in contrast to sparse coding, which requires a large number of neurons, only a very few of which would be active at a given time to encode a stimulus. Thus, different levels of inhibition between the superficial and deep layers may dictate the computations performed by a pyramidal cell depending on its postsynaptic targets (Apicella et al., 2012; Harris and Mrsic-Flogel, 2013).

Together, these results indicate that even though an excitation/inhibition balance is maintained within a layer (Pouille et al., 2009; Xue et al., 2014), this balance, i.e., the relative amounts of excitation and inhibition, may vary between different layers. The difference in the selectivity and sparseness of neuronal responses between the superficial and deep layers, as observed *in vivo* (Niell and Stryker, 2008; Sakata and Harris, 2009; O’Connor et al., 2010) is likely to emerge, at least partly, from the differential targeting of inhibitory and excitatory neurons in the different layers by long-range inputs (Yang et al., 2013; D’Souza et al., 2016), with both feedforward and feedback inhibitory motifs presumably playing important, distinct roles in controlling the gain and preserving the fidelity of signal transmission. In addition to the layer-specific, long-range excitation of inhibitory interneurons, inhibition to excitatory and inhibitory neurons from sources within an area also exhibits a laminar profile, with each neuron receiving inhibition from sources in multiple layers, and not just from neighboring interneurons (Xu and Callaway, 2009; Kätzel et al., 2011; Xu et al., 2016). Further, the recruitment of inhibition within the different layers depends not only on the laminar location of neuronal cell bodies, but also on the precise locations of inhibitory synaptic inputs along the dendrites of neurons that can traverse multiple layers (Kawaguchi and Kondo, 2002; Palmer L. et al., 2012; Muñoz et al., 2017).

The higher levels of inhibitory recruitment in the superficial layers may underlie the distinct frequency channels through which feedforward and feedback communication is achieved in the human and non-human primate brains (Bastos et al., 2015; Michalareas et al., 2016). By recording local field potentials using electrocorticography in monkeys, and by using magnetoencephalography in humans, these studies showed that feedforward pathways utilize the higher frequency gamma oscillations (40–90 Hz), while feedback pathways use slower (7–17 Hz) oscillations, to mediate long-range communication. Gamma-band synchronization is largely localized in superficial layers whereas slower oscillations predominate in deeper cortical layers (Maier et al., 2010; Buffalo et al., 2011; Roberts et al., 2013), consistent with the laminar separation of feedforward and feedback afferents. These results, taken together with the crucial role that fast-spiking interneurons play in the generation of gamma rhythms (Hasenstaub et al., 2005; Cardin et al., 2009) and the previously described laminar segregation of excitation/inhibition balances, suggest a central role of local PV+ interneurons (virtually all of which show a fast-spiking, non-adapting physiology; Chattopadhyaya et al., 2004; Hu et al., 2014) in regulating long-range communication. The observations from these studies imply that the divergent functions of feedforward and feedback pathways are accomplished not only by the laminar separation of afferents, but also by the differential recruitment of interneurons in different layers, and the subsequent induction of pathway- and layer-specific oscillations.

## Disinhibitory Circuits in Neocortex

In addition to feedforward inhibition through the recruitment of PV+ interneurons, a commonly observed long-range circuit motif is the disinhibition of pyramidal cells through the excitation of GABAergic interneurons that express the vasoactive intestinal peptide (VIP). VIP+ interneurons strongly, and with high probability, inhibit somatostatin (SST)-positive interneurons, which themselves inhibit pyramidal cells (Pfeffer et al., 2013; Jiang et al., 2015). In this way, excitation of VIP+ interneurons can “release” pyramidal cells from inhibition. Such a disinhibitory mechanism was shown to be employed by the cingulate cortex in modulating the responses of V1 neurons so that the latter’s responses to preferred orientations of visual stimuli were enhanced, while responses to non-preferred orientations were unchanged (Zhang et al., 2014). The disinhibitory circuit motif was also observed in the pathway connecting mouse primary vibrissal motor cortex to barrel cortex (Lee et al., 2013), and is thought to be a general mechanism for providing an additional layer of neuronal gain control by interareal connections throughout the neocortex (Pi et al., 2013; Muñoz et al., 2017).

The importance of interneurons in mediating long-range communication is further evidenced by the behavioral state-dependent modulation of visual cortex. During locomotion, the gain of V1 pyramidal cells in response to visual stimulation is enhanced (Niell and Stryker, 2010; Polack et al., 2013; Saleem et al., 2013; Reimer et al., 2014), which is accompanied by an increase in the firing frequency of local VIP+ interneurons as well (Fu et al., 2014; Reimer et al., 2014; Jackson et al., 2016). At first glance, this is consistent with the disinhibitory function of VIP+ interneurons. However, confounding this notion is the observation that during locomotion, the activity of SST+ interneurons was also enhanced during visual stimulation instead of being inhibited (Polack et al., 2013; Pakan et al., 2016). A possible explanation for this discrepancy is that the modulation of SST+ interneurons is context-dependent; their responses during locomotion depended on whether the task was performed during visual stimulation or in darkness (Pakan et al., 2016), indicating that the enhancement in the gain of V1 pyramidal cells during locomotion is not simply due to disinhibition, but may involve the actions of neuromodulators or the effects of locomotion in subcortical structures like the thalamus (Erisken et al., 2014; Saleem et al., 2017), which would contribute to increased gain by thalamocortical inputs (Pakan et al., 2016).

Together, these studies have demonstrated that the cortex employs a number of circuit motifs, including the long-range recruitment of PV+ and VIP+ interneurons to respectively inhibit and disinhibit local pyramidal cells, depending on context and the task the animal has to perform.

## An Organizing Role of Cortical Layer 1

As the primary target of feedback pathways, particularly in primary sensory areas, neocortical layer 1 holds a unique position in understanding the hierarchical function of the laminar layout of the cortex. Characterized by a distinct paucity of neurons, this layer is a dense neuropil of axons and dendrites that lacks the cell bodies of pyramidal cells and PV+ interneurons, but contains cell bodies of other families of GABAergic interneurons, including those that can be identified by their respective expression of calretinin, SST and/or VIP (Hestrin and Armstrong, 1996; Gonchar et al., 2007; Rudy et al., 2011; Muralidhar et al., 2013). Within the layer, long-range projecting axons from other cortical areas as well as from thalamus make excitatory contacts with dendrites of neurons residing in the layers below, notably the apical dendrites of pyramidal cells (Shipp, 2007; Cruikshank et al., 2012; Yang et al., 2013; Cruz-Martín et al., 2014; D’Souza et al., 2016). The connections formed by these afferents make up the vast majority of excitatory synapses in layer 1 (>90% in cat V1; Binzegger et al., 2004), pointing to a functionally important role of this layer as a hub for selectively integrating cortico-cortical and thalamocortical inputs (Rubio-Garrido et al., 2009; Sherman and Guillery, 2011; Larkum, M. 2013; Ji et al., 2015; Roth et al., 2016). It is important to note that neocortical layer 1 is not merely a site for feedback connections but is also an explicit target of first and higher order thalamic nuclei (Jones, 1998; Rubio-Garrido et al., 2009) as well as of feedforward projections between higher (non-primary) areas of a cortical hierarchy (Coogan and Burkhalter, 1993; D’Souza et al., 2016). As touched upon earlier, layer 1 of mouse V1 receives thalamic inputs from the dorsal lateral geniculate nucleus and from LP, each of which provides distinct visual and locomotion-related information to V1 (Cruz-Martín et al., 2014; Roth et amplification of feedforwardal., 2016).

Excitatory inputs to distal regions of a pyramidal neuron’s apical dendrite could be argued to have only minimal effects on spike generation at the axon because of substantial attenuation of the signal as it propagates to the cell body (Stuart and Spruston, 1998). However, stimulation of the apical dendrite, either antidromically or synaptically, can result in a spatially restricted influx of calcium and the generation of calcium-dependent regenerative potentials (“Ca^2+^ spikes”) in the apical dendrite (Amitai et al., 1993; Yuste et al., 1994; Schiller et al., 1997; Larkum and Zhu, 2002). The triggering of Ca^2+^ spikes provides for a putative mechanism through which coincident or strong synaptic inputs to the apical dendrite can result in long-lasting, high frequency bursts of sodium action potentials in the soma and axon (Larkum and Zhu, 2002; Williams and Stuart, 2002), which is dependent on backpropagation of the somatic action potential into the apical dendrite (Larkum et al., 2001). This has led to the proposition that a putative cellular mechanism through which top-down influence on signal propagation can be achieved is through the coincidence of a backpropagating action potential with a Ca^2+^-dependent plateau potential caused by feedback synaptic input to distal regions of the dendrite in layer 1, resulting in a context-dependent, behaviorally relevant amplification of feedforward input through Ca^2+^ spike generation (Larkum et al., 2004; Larkum, M. 2013; Takahashi et al., 2016).

The importance of excitatory inputs in layer 1 necessitates the regulation of their timing and efficacy by inhibition. The most likely candidates responsible for inhibitory control within layer 1 are the interneurons residing within the layer itself (Letzkus et al., 2011; Wozny and Williams, 2011; Jiang et al., 2013) as well as interneurons in the lower layers, such as the SST-expressing Martinotti cells, that project their axons into layer 1 (Kapfer et al., 2007; Silberberg and Markram, 2007; Gentet et al., 2012; Palmer L. et al., 2012). In rat sensorimotor cortex, at least two populations of layer 1 interneurons were shown to be able to differentially control the excitation of both layer 2/3 and layer 5 pyramidal cells through distinct monosynaptic and disynaptic networks (Jiang et al., 2013; Larkum, M. E. 2013; Lee et al., 2015), thus providing a multilayered regulation of cortical output.

In addition to being the site of electrically remote dendritic regions of the underlying neurons, a number of studies indicate that layer 1 itself may be anatomically partitioned into sub-regions, pointing to an additional computational strategy for modulating the responses of neurons in the deeper layers (Ichinohe and Rockland, 2002; Rubio-Garrido et al., 2009; Ji et al., 2015). In mouse V1, layer 1 and superficial regions of layer 2/3 exhibit a non-uniform pattern of repeating zones that strongly express the M2 acetylcholine receptor (Ji et al., 2015). These *patches* interdigitate with zones termed *interpatches* that have a significantly lower level of M2 expression. The patches and interpatches appear to play a spatial organizing role for neurons displaying different spatiotemporal preferences. The proportion of neurons that selectively responded to varying orientations, directions, speeds and motion coherence (measured by varying the proportion of stimulus dots moving in a particular direction) of visual stimuli was significantly different in regions lying directly below the M2-rich patches and those aligned with M2-weak interpatch zones (Ji et al., 2015). Further, the patches were a preferred target for a number of long-range pathways, including the dorsal lateral geniculate nucleus, and the higher areas LM and AL (see also Rubio-Garrido et al., 2009). This architecture is reminiscent of the honeycomb-like pattern observed at the border of layers 1 and 2 of rat visual cortex (Ichinohe et al., 2003). These “honeycombs”, like the patches of mouse V1, were argued to be selectively targeted by putative thalamocortical projections, but in addition, were also shown to alternate with zinc-enriched putative cortico-cortical projections (Ichinohe et al., 2003). It is reasonable to hypothesize, therefore, that the interpatch regions of mouse V1 are also a preferred target of yet unidentified cortico-cortical projections. Such an organization of alternating, adjacent regions containing circuits with distinct functions would allow for parallel, intercommunicating representations of diverse aspects of visual stimuli while preserving the retinotopic layout within V1.

It is tempting to think of the modular organization of mouse V1 as being analogous to cortical columns of higher mammals. However, there are some important differences. Unlike V1 of primates and cats, in which neurons form orientation columns that span multiple layers (Hubel and Wiesel, 1963, 1968), neurons in mouse V1 that have similar orientation preferences are randomly organized, a pattern that has been described as “salt-and-pepper” (Ohki et al., 2005). While mouse V1 pyramidal neurons that show similar visual preferences are more likely to connect with each other (Ko et al., 2011; Cossell et al., 2015), their physical positions do not appear to be organized in any columnar fashion. Interestingly, however, the M2-based patch and interpatch system was found to also exist in monkey V1, with cytochrome oxidase-rich blobs coinciding with the interpatch regions (Ji et al., 2015). This is particularly fascinating because neurons in monkey V1 within blobs are less orientation-selective than those outside blobs (Livingstone and Hubel, 1984), consistent with the demonstration that neurons aligned with interpatches in mouse V1 are less likely to be orientation-selective than those underlying patches (Ji et al., 2015). Therefore, given the relatively small size of each M2-patch and interpatch zone, it appears that this evolutionarily conserved modular system in V1 is important for the hierarchical, distributed processing of diverse visual stimulus properties within a point image.

## Summary and Concluding Remarks

The layered cortical network provides a framework to identify the fundamental connectivity rules and organizing principles by which the brain integrates internally generated cortical activity and incoming sensory stimulus-encoding signals in order to make sense of, and navigate through, the environment. In addition to stereotypic neuronal connections between layers (Thomson and Bannister, 2003; Douglas and Martin, 2004), each layer is a selective target for a variety of long-range connections whose origins include other cortical areas, thalamus, as well as the contralateral hemisphere (Shipp, 2007; Palmer L. M. et al., 2012; Hooks et al., 2013; Harris and Shepherd, 2015). Interareal cortical connections have often broadly been classified as being feedforward, feedback, or lateral, each with distinct structural and functional properties. However, observations from several studies compel us to take a more nuanced view in understanding cortico-cortical communication. The patterned targeting of layer 1 of V1 by thalamocortical afferents (Rubio-Garrido et al., 2009; Ji et al., 2015), and the examination of feedforward connections between higher visual areas (Coogan and Burkhalter, 1993; D’Souza et al., 2016), lead to the conclusion that layer 1 is not simply a target of feedback projections but also receives input from local and feedforward-projecting pyramidal cells. Further, both feedforward and feedback pathways form circuits comprising both driver-like and modulator-like synaptic connections that originate in all layers (barring layer 1; Covic and Sherman, 2011; De Pasquale and Sherman, 2011). The brain therefore utilizes a gradient of feedforward and feedback properties, both structural and cellular, depending on the hierarchical level of the interconnecting areas. This is analogous to the gradient in the excitation/inhibition balance (D’Souza et al., 2016) as well as in the proportion of supragranular neurons that project in a particular direction (Barone et al., 2000; Markov et al., 2014) across the cortical hierarchy. The excitation of apical dendrites in layer 1 as a way to amplify excitatory inputs to proximal dendrites, through the generation of Ca^2+^ spikes, may be a general mechanism employed in the cortex, albeit most commonly by feedback projections (Phillips, 2017). A system in which feedforward and feedback afferents share their “driving” and “modulating” responsibilities has important implications for our understanding of top-down control of feedforward signals because it indicates that anatomically defined feedforward and feedback pathways can each play a role in the selection and amplification of signals from the other pathway, consistent with the notion that hierarchies do not define a strict order of areas but instead depend on sensory modality (Chaudhuri et al., 2015).

The fine-scale patchy organization of receptors and/or neurites observed not only in visual cortex but also in auditory, retrosplenial and medial entorhinal cortices (Ray et al., 2014; Ji et al., 2015) likely reflects a generalized strategy of segregating parallel pathways that process distinct sensory and motor signals while also preserving topography. In the visual system, having multiple modules within the point image (Ji et al., 2015) may enable cross-talk between neighboring pyramidal cells encoding diverse spatiotemporal information. The patchy organization of layer 1 also implies that feedback projections do not act in a diffused and generic manner across a lower area but selectively modulate the activity of individual pyramidal cells depending on the subnetwork (module) to which it belongs.

## Author Contributions

RDD and AB reviewed literature and wrote the manuscript.

## Conflict of Interest Statement

The authors declare that the research was conducted in the absence of any commercial or financial relationships that could be construed as a potential conflict of interest.
